# Buying inferior to selling: Explore the impact of transaction direction on the effects of related-party transactions

**DOI:** 10.1371/journal.pone.0310767

**Published:** 2024-09-19

**Authors:** Shuang Li, Jie He

**Affiliations:** School of Business Administration, Southwestern University of Finance and Economics, Chengdu, China; PLoS ONE, UNITED STATES OF AMERICA

## Abstract

Throughout, the effects of related-party transactions (RPTs) have been a hot topic in financial markets and corporate governance research. This paper analyzes the theoretical foundation of the effects of RPTs and constructs a new indicator, the quasi-profit margin, to assess the effects of RPTs by studying their impact on the quasi-profit margin. Based on the information asymmetry between transaction parties and the information screening theory, the paper proposes the buying inferior to selling theory, systematically explaining the impact of transaction direction on the effects of RPTs. Subsequently, using panel data from Chinese A-share listed companies from 2016 to 2021, the paper constructs fixed-effects models and conducts empirical studies from both exogenous and endogenous perspectives, employing estimation methods such as high dimensional fixed effects method, two-stage least squares method, and three-stage least squares method. The research indicates that RPTs of Chinese A-share listed companies generally exhibit a tunneling effect, and the transaction direction significantly affects the effects of RPTs. The higher the proportion of RPTs conducted as sellers to the total RPTs, the smaller the overall tunneling effect of the RPTs. This study has implications for reducing the tunneling risk of RPTs and improving corporate governance for listed companies, as well as providing some references for financial regulatory authorities to identify and rectify illegal RPTs.

## Introduction

The 2023 report by the State Council of China on the state of financial affairs emphasized the need to comprehensively strengthen and improve financial regulation, resolutely correct illegal related-party transactions (RPTs), and focus on maintaining the stable operation of financial markets. In emerging economies represented by China, RPTs have always been an important component of market economic activities. In recent years, with the continuous optimization and upgrading of market mechanisms, the external market has become increasingly functional, but the internal market has not shown a significant contraction. RPTs occurring in the internal market still account for a considerable proportion of market economic activities. In the A-share market in China, the average proportion of annual RPTs to total assets at the beginning of the year for listed companies in 2021 was as high as 31.7%.

Existing literature generally posits that RPTs exhibit two effects: "efficiency enhancement" and "tunneling" [1,2]. The existence of internal markets and the occurrence of RPTs were initially intended to complement the incompleteness of external markets [1,3], improve resource allocation efficiency [4], ensure smooth and efficient transactions, promote the prosperity and stability of financial markets, and drive the rapid development of a market-oriented economy. Therefore, in a certain sense, RPTs have a support effect on the development of listed companies and the enhancement of enterprise performance [5]. However, more research shows that RPTs have a more significant tunneling effect. Some scholars believe that from the perspective of capital allocation efficiency, internal capital markets are inefficient [6–9]. Internal capital markets have gradually alienated into a channel for major shareholders to seize company benefits [10,11], and RPTs have become a device for major shareholders to infringe on the interests of minority shareholders [12–14]. The tunneling effect of RPTs can severely damage the interests of listed companies, hinder their healthy development, and even pose a significant threat to the stability of regional financial markets [15].

What factors influence the effects of RPTs? Scholars have conducted extensive research from various perspectives, including firm size and ownership structure [16], equity structure [17], independent directors [18], board characteristics [19], audit quality [20], analyst coverage [21], financial regulatory policies [22], and dividend policies [23]. However, no literature has yet examined the impact of transaction direction on the effects of RPTs, a research perspective that has been overlooked in existing studies.

In any direct transaction, there are two roles: the seller and the buyer. According to the information asymmetry theory, the information between buyers and sellers is asymmetrical, which directly affects their transaction costs and outcomes. Similarly, in RPTs, there are seller and buyer roles. Does the difference in transaction roles, i.e., transaction direction, influence the effects of RPTs? This question holds significant research value both theoretically and practically. It not only helps clarify the influencing factors of the effects of RPTs, expanding the theoretical framework and enriching related research literature, but also provides insights for listed companies to mitigate the tunneling risk of RPTs and enhance corporate governance. Additionally, it offers some references for financial regulatory authorities in identifying and addressing illegal RPTs.

This paper first conducts theoretical analysis for the efficiency enhancement and tunneling effects of RPTs based on the transaction cost theory and the information asymmetry theory. It then constructs a new indicator, the quasi-profit margin, to evaluate the effects of RPTs by studying their impact on the quasi-profit margin. Furthermore, based on the information asymmetry between the transaction parties and the information screening theory, the paper proposes the buying inferior to selling theory, systematically elucidating the impact of transaction direction on the effects of RPTs. Subsequently, using panel data of Chinese A-share listed companies from 2016 to 2021, the paper constructs fixed effects models and conducts empirical research from both exogenous and endogenous perspectives, employing estimation methods such as high dimensional fixed effects method, two-stage least squares method, and three-stage least squares method.

The main innovations and potential marginal contributions of this paper are as follows:

In terms of research direction, this paper focuses on exploring the impact of transaction direction on the effects of RPTs, a research perspective that has been somewhat neglected in existing literature, thereby possessing a certain degree of novelty.

In terms of research method, (1) this article proposes a new method to measure the effects of RPTs, that is, to use the quasi-profit margin to characterize the comprehensive changes in revenue, costs, and expenses of listed companies, and to measure the effects of RPTs by studying their impact on the quasi-profit margin. (2) In terms of research design and model construction, this article does not persist in trying to directly measure the direct effects of RPTs conducted from the seller or buyer perspective. Instead, it takes a different approach and uses the proportion of RPTs conducted from the seller perspective to the total amount of RPTs as a moderating variable, and constructs a moderation effect model to study the impact of transaction direction on the effects of RPTs. The research methods of this article may provide some inspiration for scholars who focus on RPTs.

Theoretically, this paper pioneeringly proposes the buying inferior to selling theory. Through theoretical exposition and empirical testing, it reveals the impact of transaction direction on the effects of RPTs, enriching the relevant research literature and expanding the theoretical framework of the influencing factors of the effects of RPTs.

Practically, this study provides some references and insights for listed companies and financial regulatory authorities. For listed companies, engaging more in RPTs as sellers can mitigate the tunneling effects and reduce the risk of asset stripping. For financial regulatory authorities, there is a need to further refine the control policies of RPTs. For example, regulatory authorities could require listed companies to disclose RPTs information categorized by transaction roles and to particularly scrutinize RPTs in which the company acts as a buyer.

The remainder of this paper is organized in the following order: literature review, theoretical analysis and hypothesis development, research design, empirical results analysis, robustness tests, conclusions and discussions.

## Literature review

### The two effects of RPTs

Is RPTs always a good or bad thing? The answer is not so simple.

Some studies have shown that RPTs have a support effect on listed companies. In emerging market economies where external markets are incomplete, internal capital markets can replace some of the functions of external markets [1,3], and play a role in relaxing financing constraints and reducing financing difficulties [24,25]. Corporate group members can improve resource allocation efficiency and obtain strategic advantages by RPTs, and consolidate their market position through cooperation, such as information sharing, fund financing, and joint investment [4]. Therefore, RPTs are beneficial to corporate value [5]. Khanna & Palepu found that internal products, labor, and capital indices of affiliated firms were positively correlated with the value of affiliated firms after studying business groups in India [1]. Chang & Hong found that RPTs within Korean corporate groups enhanced the value of member companies [26]. Perotti & Gelfer found that members of Russian financial business groups could allocate capital more effectively through RPTs than external capital markets [27]. Wang, Chen & Li empirically found that related-party guarantees could significantly reduce the financing constraints of listed companies [28]. Therefore, in a sense, RPTs are an institutional arrangement that helps transactions and reduces transaction costs [1,29].

However, more research shows that RPTs have a more significant tunneling effect. Some scholars believe that from the perspective of capital allocation efficiency, internal capital markets are inefficient [6–9]. Internal capital markets have gradually alienated into a channel for major shareholders to seize company benefits [10,11], and RPTs have become a device for major shareholders to infringe on the interests of minority shareholders [1]. El-Helaly, Georgiou & Lowe argue that RPTs provide opportunities for wealth transfer between listed companies and their related parties, gradually becoming a tool for shareholders to extract wealth [20]. Baek, Kang & Lee found that related-party mergers and acquisitions between Korean listed companies and other members within the group would lead to a drop in the former’s stock prices, during which major shareholders seized the interests of external minority shareholders [30]. Bertrand, Mehta & Mullainathan found that there may be profit transfer within Indian business groups [31]. Li, Sun & Wang found that controlling shareholders of Chinese listed companies may occupy the funds of listed companies through RPTs, causing the interests of minority shareholders to be damaged [32]. Chang [33] and Cheung, Rau & Stouraitis [34] found that major shareholders used RPTs to strip listed companies. Dou & Lu found that the value of cash holdings of listed companies with a high proportion of related-party deposits was lower [35]. Tang & Han found that agency problems are more severe in related party mergers and acquisitions [36]. Alhadab, Abdullatif & Mansour found that RPTs are negatively correlated with accrual earnings management in listed industrial companies in Jordan [17]. Ismail, El-Deeb & Halim’s research based on Egyptian listed companies found that RPTs are negatively correlated with corporate fair value and that RPTs significantly moderate the impact of political connections on corporate fair value [37].

Overall, RPTs have both support and tunneling effects. The dominant effect, however, varies across different time periods, intervals, cross-sections, and comprehensive factors of internal and external governance mechanisms. This effect is not fixed and may differ depending on the circumstances [1,2]. Thus, a dialectical and objective perspective should be taken when considering the effects of RPTs.

### The measurement of the effects of RPTs

There are many types of RPTs, so what indicators and methods should be adopted to measure the effects of RPTs?

In existing literature, some scholars equate RPTs with tunneling, and use indicators such as the ratio of RPTs to revenue [38] or the natural logarithm of the total amount of RPTs [4] to directly measure the tunneling effect of RPTs. This paper refers to this method as the "overall indicator direct measurement method". Other scholars use indicators such as related party funds utilization [32], related party guarantees [39], or related party deposits [35] to directly measure the tunneling effect of RPTs. This paper refers to this method as the "sub-indicator direct measurement method". Some scholars indirectly measure the effects of RPTs by studying the impact of related sales on gross profit margins or related purchases on the main business cost rate [40]. This paper refers to this method as the "sub-indicator indirect measurement method". Additionally, some scholars use market reactions or performance changes within a certain time window after RPTs occur as substitute indicators to measure the effects of RPTs [12,41]. This paper refers to this method as the "substitute indicator measurement method".

This paper believes that all these measurement methods have certain limitations. The limitations of the "overall indicator direct measurement method" and the "sub-indicator direct measurement method" lie in their narrow focus on the tunneling effect of RPTs, which may lead to biased conclusions and harm innocent parties. The limitations of the "sub-indicator direct measurement method" and the "sub-indicator indirect measurement method" lie in their research focus on a specific type of RPTs, which may fail to evaluate the overall effect of various types of RPTs in listed companies from a global perspective. This may lead to not seeing the forest for the trees. The "substitute indicator measurement method" suffers from the problem of large data noise and poor result robustness.

### The factors affecting the effects of RPTs

In existing literature, scholars have studied the factors influencing the effects of RPTs from internal and external perspectives.

Internally, Alhadab, Abdullatif & Mansour evaluated the effects of RPTs on earnings management and examined the influence of ownership structure on the effects of RPTs [17]. Chen et al. explored the impact of chairman’s political positions and independent directors on the effects of RPTs [18]. Du, Li & Wang found that the firm size and ownership structure have significant influence on the effects of RPTs [16]. Gavana, Gottardo & Moisello empirically studied the influence of board characteristics on the effects of RPTs using non-financial listed companies in Italy [19]. Additionally, Ryngaert & Thomas investigated how pre-existing relationships between listed companies and related parties versus post-relationship transactions affect RPTs, showing distinctly different effects in each case [42].

Externally, El-Helaly, Georgiou & Lowe researched the impact of audit quality on the effects of RPTs [20]. Liu, Zhang & Dai found that analyst coverage mitigates information asymmetry and the regulatory effects positively impacting the effects of RPTs [21]. Beaver et al. studied the effects of regulatory policies such as approval and disclosure requirements on the effects of RPTs [22]. Kong, Ji & Liu discovered that mandatory dividend policies affect the effects of RPTs, where these transactions promote resource allocation efficiency particularly under adverse external cash shocks to listed companies [23].

## Theoretical analysis and hypothesis development

### The effects of RPTs

#### The theoretical basis of the two effects of RPTs

Existing literature generally acknowledges that RPTs have two main effects: "efficiency enhancement" and "tunneling" [1,2]. What are the theoretical foundations underlying these two effects?

According to transaction cost theory [43], market transactions typically incur various costs including search costs, information costs, bargaining costs, decision costs, monitoring costs, and default costs [44]. Reducing transaction costs helps improve transaction efficiency, thereby promoting corporate development. Compared to arm’s length transactions, RPTs involve close relationships between the listed company and its related parties. This closeness helps reduce costs associated with searching for product information and transaction partners, exchanging information between parties, and bargaining costs. This reduction in transaction costs and associated expenses leads to the efficiency-enhancing effect of RPTs.

Based on agency theory [45] and information asymmetry theory [46], large shareholders of listed companies and small shareholders are in an agency relationship where information asymmetry exists. Large shareholders, having greater influence and more information about the company’s management, decisions, and operations, are in an advantageous position with superior information. In contrast, small shareholders have less information and weaker influence. Large shareholders may exploit this information asymmetry to extract benefits from small shareholders, known as tunneling. RPTs typically occur between listed companies and entities controlled by large shareholders, where small shareholders, already disadvantaged in terms of information and influence, find it difficult to fully understand and evaluate the specifics of RPTs. This creates opportunities for large shareholders to exploit RPTs for their own gain, thereby constituting the tunneling effect of RPTs.

#### How to measure the effects of RPTs

The article argues that RPTs directly or indirectly affect the operating income, cost, or expenses of listed companies. The change in operating income, cost, or expenses caused by RPTs can intuitively reflect the effects of RPTs. To characterize the comprehensive changes in the operating income, cost, and expenses of listed companies, this article constructs a new variable called "quasi-profit margin". The calculation method is as follows:

Quasi-profit margin = (Main operating profit—Selling expenses—Financial expenses—Management expenses) / Main Operating revenue

Quasi-profit margin is economically similar to profit margin, but it is distinct from indicators such as operating profit margin, net profit margin, EBIT margin, and EBITDA margin. Therefore, this new variable is named "quasi-profit margin" in this article.

Decomposing the quasi-profit margin reveals that it equals the difference between the main operating profit margin and the period expense rate. From the theoretical analysis, if RPTs exhibit an efficiency-enhancing effect, they can increase the main operating profit margin by reducing the operating costs of the listed company, or they can lower the period expense rate by reducing expenses incurred during sales and management processes, thereby enhancing the quasi-profit margin. Conversely, if RPTs show tunneling effects, they directly reduce the main operating profit margin of the listed company or increase the period expense rate, thereby decreasing the quasi-profit margin.

According to the meaning of quasi-profit margin above, if RPTs are significantly positively correlated with quasi-profit margin, it indicates that RPTs have caused a positive comprehensive change in the operating income, cost, and expenses of listed companies, which reflects a support effect. Conversely, if RPTs are significantly negatively correlated with quasi-profit margin, it indicates that RPTs have caused a negative comprehensive change in the operating income, cost, and expenses of listed companies, which reflects a tunneling effect.

To verify the relationship between RPTs and quasi-profit margin, that is, to test the average effect of RPTs from a whole sample perspective, this article proposes the following hypotheses:

***Hypothesis 1*:** RPTs have a significant impact on quasi-profit margin.
***Hypothesis 1–1*:** RPTs are significantly negatively correlated with quasi-profit margin, indicating that RPTs have an average tunneling effect.
***Hypothesis 1–2*:** RPTs are significantly positively correlated with quasi-profit margin, indicating that RPTs have an average support effect.

### The impact of transaction direction on the effects of RPTs

#### Two transaction roles

In any direct transaction, there are two transaction roles: the seller and the buyer. In the same transaction, both parties play the role of the seller and the buyer, respectively. Transaction role is not fixed and can vary for the same company in different transactions.

Similarly, in RPTs, there are also two transaction roles: the seller and the buyer. In the same RPT, the listed company and the related party play the roles of the seller and the buyer, respectively. All RPTs of each listed company can be divided into two parts according to the transaction roles, that is, RPTs conducted from the perspective of the seller and those conducted from the perspective of the buyer.

#### Seller’s advantage principle

According to the theory of asymmetric information [46], the two parties in a transaction, namely the seller and the buyer, possess asymmetric information. Compared to the buyer, the seller holds an informational advantage. In the transaction process, the seller provides the goods while the buyer provides the demand. The demand-providing buyer is downstream in economic activities, while seller of goods, as producers or salespeople, are upstream or midstream. The seller is more knowledgeable about the raw materials, production process, processing technology, various additional costs and fees, product performance, market trends, and other information.

According to the theory of information screening [47,48], in order to mitigate the adverse effects of asymmetric information on themselves, buyers typically engage in information screening of the information provided by sellers. This screening process inadvertently increases the transaction costs for the buyer, resulting in higher transaction costs borne by the buyer compared to the relatively lower costs borne by the seller in the same transaction.

#### Buying inferior to selling theory

This article posits that RPTs between listed companies and their related parties are also conform to the seller’s advantage principle. This leads to the transaction direction may influences the effects of RPTs and the direction of interest flow in the trading market. The following will discuss this from three dimensions.

1. Dimension of a single RPT
  In a single RPT, the listed company and the related party respectively act as the seller and the buyer. The one who takes the seller’s role has the seller’s advantage. Ignoring the impact of external environmental changes and only considering the intrinsic nature of the transaction, the party with the seller’s advantage, namely the seller, is the benefit input side, while the one without the seller’s advantage, namely the buyer, is the benefit output side. The seller’s advantage determines the most basic direction of interest flow in the trading market.
2. Dimension of multiple RPTs
  A listed company may have multiple RPTs with various related parties, with different transaction roles in each RPT. The multiple RPTs of each listed company can be divided into two parts according to the transaction role: RPTs carried out in the seller’s role and those carried out in the buyer’s role. According to the "seller’s advantage" principle, the greater the proportion of RPTs in which the listed company takes the seller’s role, the greater the average seller’s advantage of the listed company compared to its related parties, and the smaller the overall tunneling effect of the RPTs of the listed company. Conversely, the smaller the proportion of RPTs in which the listed company takes the seller’s role, the smaller the average seller’s advantage of the listed company compared to its related parties, and the greater the overall tunneling effect of the RPTs of the listed company.
3. Dimension of coexistence of RPTs and non-RPTs
  A listed company may engage in both RPTs with various related parties and non-RPTs with other non-related parties. Both RPTs and non-RPTs are games that conform to the "seller’s advantage" principle. If we consider the transaction roles of seller and buyer and discuss the RPTs and non-RPTs of the listed company together, we can find that the greater the proportion of RPTs in which the listed company takes the seller’s role, the smaller the proportion of non-RPTs in which the listed company takes the seller’s role or the greater the proportion of non-RPTs in which the listed company takes the buyer’s role. This means that the overall direction of interest flow among the listed company, its related parties, and non-related parties is from the related parties to the listed company, and then from the listed company to the non-related parties. Conversely, the smaller the proportion of RPTs in which the listed company takes the seller’s role, the greater the proportion of non-RPTs in which the listed company takes the seller’s role or the smaller the proportion of non-RPTs in which the listed company takes the buyer’s role. This means that the overall direction of interest flow among the listed company, its related parties, and non-related parties is from non-related parties to the listed company and then from the listed company to its related parties.

The above content is referred to as the buying inferior to selling theory in this paper. In order to verify the impact of the transaction direction on the effects of RPTs from a full sample perspective, i.e., to verify the buying inferior to selling theory proposed in this paper, the following hypotheses are proposed:

***Hypothesis 2*:** The transaction direction has a significant impact on the effects of RPTs.
***Hypothesis 2–1*:** The greater the proportion of RPTs conducted from the seller role, the smaller the tunneling effect of RPTs.
***Hypothesis 2–2*:** The greater the proportion of RPTs conducted from the seller role, the larger the tunneling effect of RPTs.

## Research design

### Sample selection and data sources

This paper takes Chinese A-share listed companies from 2016 to 2021 as the research objects and selects the sample data using the following criteria: (1) excluding samples from the financial industry; (2) deleting samples from ST companies; (3) removing samples with abnormal or missing data. Finally, 3561 companies with a total of 16433 valid observation samples of "company-year" were obtained, as shown in Table 1. In order to eliminate the influence of extreme values on the research conclusions, this paper performs winsorization trimming on all continuous variables at the upper and lower 1% levels. The sample data in this study mainly comes from the China CSMAR database.

**Table 1:** Summary of sample distribution.

*Year*	*Samples*	*Percent*	*Cum*.
*2016*	*2148*	*13*.*07*	*13*.*07*
*2017*	*2348*	*14*.*29*	*27*.*36*
*2018*	*2731*	*16*.*62*	*43*.*98*
*2019*	*2834*	*17*.*25*	*61*.*22*
*2020*	*2993*	*18*.*21*	*79*.*44*
*2021*	*3379*	*20*.*56*	*100*.*00*
*Total*	*16433*	*100*.*00*	

### Variable definitions

#### Dependent variable

This paper employs the variable named "quasi-profit margin" as the dependent variable for empirical research, which is denoted by the symbol *QPM* in this paper. See Chapter “How to measure the effects of RPTs” for the detailed meaning and calculation process of quasi-profit margin.

#### Explanatory variables

1. RPTs
  The annual total amount of RPTs of listed companies is measured in relation to their total assets at the beginning of the year using the ratio, and it is denoted by the symbol *RPTs*.
2. Transaction direction
  This article measures the transaction direction of listed companies in RPTs by the ratio of the amount of RPTs conducted by the listed companies as sellers to the total amount of RPTs, and it is denoted as *SELLER*.

#### Control variables

1. Continuous Variables
  Drawing on the practices in the existing literature in this research area, this study employs continuous variables such as company size (*SIZE*), leverage (*LEV*), equity concentration measured by the shareholding proportion of the largest shareholder (*TOP*1), the degree of equity balance measured by the ratio of the sum of the shareholding ratios of the second to fifth largest shareholders to that of the largest shareholder (*BALANCE*), main business revenue growth rate (*MG*), composite market beta value (*Betavals*), tradable share proportion (*Tradeable*), company age (*ESTAGE*), and top three executive compensation (*MPAY*) as control variables.
2. Discrete Variables
  Drawing on existing literature in the research field, this article employs discrete variables such as nature of ownership (*SOE*), whether the auditor comes from one of the Big four accounting firms (*BIG*4), and type of audit opinion (*AUDITTYP*) as control variables.

The main variable definitions and descriptions of this article are shown in Table 2.

**Table 2:** Variable definitions and descriptions.

Variable Types	Variable Symbol	Variable Definition	Variable Description
**Dependent variable**	*QPM*	Quasi-profit margin	*QPM* = (main operating profit—selling expenses—financial expenses—administrative expenses) / main operating revenue.
**Explanatory variable**	*RPTs*	Related-party transactions	*RPTs* = Annual total amount of related-party transactions (in RMB) / Total assets at the beginning of the year (in RMB).
	*SELLER*	Transaction direction	The ratio of the amount of RPTs conducted by the listed companies as sellers to the total amount of RPTs.
**Control variables**	*SIZE*	Company size	Natural logarithm of total assets at the beginning of the year.
	*LEV*	Leverage	The ratio of total debt to total assets
	*TOP1*	Equity concentration	the shareholding proportion of the largest shareholder of the listed company
	*BALANCE*	The degree of equity balance	the ratio of the sum of the shareholding ratios of the second to fifth largest shareholders to that of the largest shareholder in listed companies
	*MG*	Main business revenue growth rate	Main business revenue growth rate is equal to (the current year-end main business revenue—the previous year-end main business revenue) divided by the previous year-end main business revenue.
	*Betavals*	Composite market beta value	Stock risk index (calculated using the comprehensive return rate of the Shanghai and Shenzhen markets as the rate of return for all stocks).
	*Tradeable*	Tradable share proportion	The ratio of the number of outstanding shares to the total number of shares.
	*ESTAGE*	Company age	Natural logarithm of (year of observation—year of establishment + 1).
	*MPAY*	Top three executive compensation	Natural logarithm of (the sum of salaries of the top three senior executives + 1).
	*SOE*	Nature of ownership	A dummy variable that takes the value of 1 if the actual controller of the listed company is of state-owned nature, and 0 otherwise.
	*BIG4*	Whether the auditor comes from one of the Big 4 accounting firms	A dummy variable that takes the value of 1 if the auditor is from one of the Big four accounting firms, and 0 otherwise.
	*AUDITTYP*	Type of audit opinion	A dummy variable that takes the value of 1 if the auditor issued a standard unqualified opinion on the company’s annual report, and 0 otherwise.
	*YEAR*	Dummy Variables for Year	A set of dummy variables representing the year of observation, with 2015 as the base year.
	*INDUSTRY*	Dummy Variables for Industry	A set of 20 dummy variables representing the industry in which the firm operates, with the manufacturing industry was further subdivided into second-level industries.

### Model design

#### The effects of RPTs

To verify the relationship between RPTs and "quasi-profit margin," that is, to test the average effect of RPTs from the perspective of the entire sample, this paper constructs the following model.


$${QPM}_{it}=\alpha_{0}+{\alpha_{1}RPTs}_{it}+\alpha_{2}{SIZE}_{it}+\alpha_{3}{LEV}_{it}+\alpha_{4}{TOP1}_{it}+\alpha_{5}{BALANCE}_{it}+\alpha_{6}{MG}_{it}+\alpha_{7}{Betavals}_{it}+\alpha_{8}{Tradeable}_{it}+\alpha_{9}{SOE}_{it}+\alpha_{10}{ESTAGE}_{it}+\alpha_{11}{BIG4}_{it}+\alpha_{12}{AUDITTYP}_{it}+\alpha_{13}{MPAY}_{it}+{YEAR}_{FE}+{INDUSTRY}_{FE}+\varepsilon$$ Model (1)

In the equation, the subscript "*it*" represents the variable data of company *i* in year *t*,*YEAR*_*FE*_ and *INDUSTRY*_*FE*_ represent year and industry fixed effects, and *ε* represents the random error term.

#### The impact of transaction direction on the effects of RPTs

To verify the impact of transaction direction on RPTs in the full sample, that is, to test the buying inferior to selling theory proposed in this paper, a moderated effect model is constructed based on Model (1) as follows:

$${QPM}_{it}=\alpha_{0}+{\alpha_{1}RPTs}_{it}+\alpha_{2}{SELLER}_{it}+\alpha_{3}{RPTs\times SELLER}_{it}+\alpha_{4}{SIZE}_{it}+\alpha_{5}{LEV}_{it}+\alpha_{6}{TOP1}_{it}+\alpha_{7}{BALANCE}_{it}+\alpha_{8}{MG}_{it}+\alpha_{9}{Betavals}_{it}+\alpha_{10}{Tradeable}_{it}+\alpha_{11}{SOE}_{it}+\alpha_{12}{ESTAGE}_{it}+\alpha_{13}{BIG4}_{it}+\alpha_{14}{AUDITTYP}_{it}+\alpha_{15}{MPAY}_{it}+{YEAR}_{FE}+{INDUSTRY}_{FE}+\varepsilon$$ Model (2)

In the equation, the subscript "*it*" represents the variable data for company *i* in year *t*,*YEAR*_*FE*_ and *INDUSTRY*_*FE*_ represent the year and industry fixed effects, and *ε* represents the random error term.

## Empirical results analysis

### Descriptive statistics and analysis

Table 3 reports the descriptive statistics of the main variables in the full sample. The minimum and maximum values for *QPM* are -0.736 and 0.528, respectively, while the minimum and maximum values for *RPTs* are 0.00117 and 3.328, respectively. These results indicate significant differences in the quasi-profit margin and RPTs between different listed companies. The average value of *RPTs* is 0.366, which suggests that, on average, RPTs account for a significant proportion of total assets (approximately 36.6%) in the sample of all listed companies. The average value of *SELLER* is 0.405, indicating that, on average, only 40.5% of RPTs are conducted from the seller’s perspective, while listed companies tend to engage in RPTs from the buyer’s perspective. The average value of *TOP*1 is 0.332, suggesting a significant concentration of equity ownership among Chinese A-share listed companies. The descriptive statistics of other variables are generally consistent with prior research and existing literature.

**Table 3:** Descriptive statistics of major variables.

*VARIABLES*	*N*	*mean*	*sd*	*min*	*p25*	*p50*	*p75*	*max*
*QPM*	*16433*	*0*.*109*	*0*.*164*	*-0*.*736*	*0*.*0422*	*0*.*107*	*0*.*188*	*0*.*528*
*RPTs*	*16433*	*0*.*366*	*0*.*530*	*0*.*00117*	*0*.*0403*	*0*.*184*	*0*.*466*	*3*.*328*
*SELLER*	*16433*	*0*.*405*	*0*.*345*	*0*	*0*.*0486*	*0*.*360*	*0*.*713*	*0*.*997*
*SIZE*	*16433*	*22*.*14*	*1*.*267*	*19*.*63*	*21*.*23*	*21*.*98*	*22*.*85*	*26*.*09*
*LEV*	*16433*	*0*.*418*	*0*.*201*	*0*.*0571*	*0*.*259*	*0*.*407*	*0*.*557*	*0*.*941*
*TOP1*	*16433*	*0*.*332*	*0*.*142*	*0*.*0845*	*0*.*222*	*0*.*309*	*0*.*423*	*0*.*740*
*BALANCE*	*16433*	*0*.*781*	*0*.*604*	*0*.*0364*	*0*.*314*	*0*.*628*	*1*.*105*	*2*.*868*
*MG*	*16433*	*0*.*193*	*0*.*472*	*-0*.*647*	*-0*.*0132*	*0*.*118*	*0*.*289*	*3*.*303*
*Betavals*	*16433*	*1*.*105*	*0*.*381*	*0*.*218*	*0*.*864*	*1*.*115*	*1*.*338*	*2*.*324*
*Tradeable*	*16433*	*0*.*789*	*0*.*242*	*0*.*101*	*0*.*623*	*0*.*890*	*1*.*000*	*1*
*ESTAGE*	*16433*	*2*.*991*	*0*.*283*	*2*.*079*	*2*.*833*	*2*.*996*	*3*.*219*	*3*.*526*
*MPAY*	*16433*	*14*.*58*	*0*.*667*	*12*.*86*	*14*.*15*	*14*.*56*	*14*.*98*	*16*.*50*
*SOE*	*16433*	*0*.*310*	*0*.*463*	*0*	*0*	*0*	*1*	*1*
*BIG4*	*16433*	*0*.*0545*	*0*.*227*	*0*	*0*	*0*	*0*	*1*
*AUDITTYP*	*16433*	*0*.*961*	*0*.*194*	*0*	*1*	*1*	*1*	*1*

#### Correlation test and analysis

Table 4 presents the results of Pearson correlation coefficient test and Spearman correlation coefficient test for the main variables in this study. The correlation coefficient test results show that: (1) explanatory variables and main control variables are significantly correlated with the dependent variable, indicating that the variable construction and selection in this study are reasonable; (2) except for the absolute value of the correlation coefficient between *TOP*1 and *BALANCE* being greater than 0.6, the correlation coefficients between other explanatory variables and control variables meet the research requirements, indicating that there is no serious problem of multicollinearity among the selected variables in this study; (3) the Pearson correlation coefficient and Spearman correlation coefficient between *RPTs* and *QPM* are both significantly negative, which preliminarily verifies Hypothesis 1 and Hypothesis 1–1 in this study; (4) the significance of the correlation coefficients between *SELLER* and *QPM*, *RPTs* is relatively high, indicating that *SELLER* may have a moderating effect on the relationship between *RPTs* and *QPM*.

**Table 4:** Pearson and spearman correlation test results for main variables.

	*QPM*	*RPTs*	*SELLER*	*SIZE*	*LEV*	*TOP1*	*BALANCE*	*MG*	*Betavals*	*Tradeable*	*ESTAGE*	*MPAY*	*SOE*	*BIG4*	*AUDITTYP*
*QPM*	*1*.*000*	*-0*.275 ***	*-0*.089 ***	*-0*.083 ***	*-0*.402 ***	*0*.084 ***	*0*.061 ***	*0*.256 ***	*0*.035 ***	*-0*.222 ***	*-0*.129 ***	*0*.194 ***	*-0*.161 ***	*0*.019 **	*0*.203 ***
*RPTs*	*-0*.153 ***	*1*.*000*	*0*.212 ***	*0*.164 ***	*0*.443 ***	*-0*.*003*	*-0*.046 ***	*0*.072 ***	*0*.030 ***	*0*.059 ***	*0*.066 ***	*-0*.059 ***	*0*.104 ***	*-0*.014 *	*-0*.074 ***
*SELLER*	*-0*.039 ***	*0*.049 ***	*1*.*000*	*0*.255 ***	*0*.133 ***	*-0*.045 ***	*-0*.035 ***	*-0*.*004*	*-0*.*003*	*0*.160 ***	*0*.072 ***	*0*.092 ***	*0*.125 ***	*0*.056 ***	*-0*.*010*
*SIZE*	*-0*.*012*	*0*.027 ***	*0*.195 ***	*1*.*000*	*0*.460 ***	*0*.119 ***	*-0*.120 ***	*-0*.047 ***	*-0*.149 ***	*0*.289 ***	*0*.190 ***	*0*.371 ***	*0*.357 ***	*0*.236 ***	*0*.*012*
*LEV*	*-0*.363 ***	*0*.307 ***	*0*.088 ***	*0*.457 ***	*1*.*000*	*0*.014 *	*-0*.072 ***	*0*.*011*	*-0*.087 ***	*0*.208 ***	*0*.146 ***	*0*.090 ***	*0*.232 ***	*0*.098 ***	*-0*.147 ***
*TOP1*	*0*.095 ***	*0*.019 **	*-0*.044 ***	*0*.171 ***	*0*.018 **	*1*.*000*	*-0*.739 ***	*0*.*006*	*-0*.076 ***	*0*.020 **	*-0*.036 ***	*0*.*002*	*0*.197 ***	*0*.107 ***	*0*.089 ***
*BALANCE*	*0*.028 ***	*-0*.028 ***	*-0*.021 ***	*-0*.109 ***	*-0*.080 ***	*-0*.694 ***	*1*.*000*	*0*.047 ***	*0*.015 **	*-0*.226 ***	*-0*.069 ***	*0*.066 ***	*-0*.246 ***	*-0*.*011*	*-0*.035 ***
*MG*	*0*.198 ***	*0*.229 ***	*-0*.023 ***	*-0*.075 ***	*0*.027 ***	*-0*.*003*	*0*.042 ***	*1*.*000*	*0*.065 ***	*-0*.179 ***	*-0*.101 ***	*0*.094 ***	*-0*.090 ***	*0*.*000*	*0*.138 ***
*Betavals*	*0*.052 ***	*0*.014 *	*-0*.*001*	*-0*.164 ***	*-0*.101 ***	*-0*.074 ***	*0*.019 **	*0*.035 ***	*1*.*000*	*-0*.202 ***	*-0*.218 ***	*-0*.112 ***	*-0*.092 ***	*-0*.073 ***	*0*.039 ***
*Tradeable*	*-0*.190 ***	*-0*.019 **	*0*.158 ***	*0*.284 ***	*0*.221 ***	*-0*.094 ***	*-0*.162 ***	*-0*.161 ***	*-0*.191 ***	*1*.*000*	*0*.302 ***	*0*.021 ***	*0*.368 ***	*0*.072 ***	*-0*.051 ***
*ESTAGE*	*-0*.113 ***	*0*.056 ***	*0*.060 ***	*0*.170 ***	*0*.147 ***	*-0*.043 ***	*-0*.055 ***	*-0*.046 ***	*-0*.216 ***	*0*.288 ***	*1*.*000*	*0*.069 ***	*0*.261 ***	*0*.034 ***	*-0*.050 ***
*MPAY*	*0*.197 ***	*-0*.076 ***	*0*.088 ***	*0*.394 ***	*0*.083 ***	*0*.016 **	*0*.063 ***	*0*.034 ***	*-0*.104 ***	*0*.037 ***	*0*.069 ***	*1*.*000*	*0*.018 **	*0*.196 ***	*0*.088 ***
*SOE*	*-0*.100 ***	*0*.050 ***	*0*.099 ***	*0*.372 ***	*0*.234 ***	*0*.207 ***	*-0*.216 ***	*-0*.060 ***	*-0*.092 ***	*0*.313 ***	*0*.248 ***	*0*.019 **	*1*.*000*	*0*.105 ***	*0*.057 ***
*BIG4*	*0*.029 ***	*-0*.025 ***	*0*.047 ***	*0*.305 ***	*0*.094 ***	*0*.120 ***	*-0*.015 *	*-0*.*002*	*-0*.069 ***	*0*.047 ***	*0*.023 ***	*0*.230 ***	*0*.105 ***	*1*.*000*	*0*.025 ***
*AUDITTYP*	*0*.310 ***	*-0*.061 ***	*-0*.*005*	*0*.023 ***	*-0*.192 ***	*0*.085 ***	*-0*.036 ***	*0*.079 ***	*0*.049 ***	*-0*.059 ***	*-0*.051 ***	*0*.096 ***	*0*.057 ***	*0*.025 ***	*1*.*000*

Note 1: The content in the upper right triangle of the table represents the results of Spearman correlation tests, while the content in the lower left triangle represents the results of Pearson correlation tests.

Note 2

"***" denotes p<0.01

"**" denotes p<0.05, and

"*" denotes p<0.1, indicating statistical significance at the 1%, 5%, and 10% levels, respectively.

### Basic regression results analysis

#### The effects of RPTs

This paper conducts regression analysis of Model (1) using LSDV, HDFE, and mixed regression with Driscoll-Kraay standard errors, and the regression results are shown in Table 5.

**Table 5:** Regression results of Model (1).

*Serials*	*(1)*	*(2)*	*(3)*
*Estimation Method*	*LSDV*	*HDFE*	*Driscoll-Kraay Pooled OLS*
*Model Type*	*Linear Model*		
*Std*. *Err*. *Type*	*Vce cluster (industry)*		*Driscoll-Kraay Std*. *Err*.
*Dependent Variable*	*QPM*		
*RPTs*	*-0*.019***	*-0*.019***	*-0*.019***
	*(-6*.*31)*	*(-6*.*31)*	*(-15*.*56)*
*SIZE*	*0*.019***	*0*.019***	*0*.019***
	*(4*.*51)*	*(4*.*51)*	*(11*.*88)*
*LEV*	*-0*.294***	*-0*.294***	*-0*.294***
	*(-17*.*91)*	*(-17*.*92)*	*(-11*.*61)*
*TOP1*	*0*.116***	*0*.116***	*0*.116***
	*(5*.*14)*	*(5*.*14)*	*(17*.*26)*
*BALANCE*	*0*.013***	*0*.013***	*0*.013***
	*(3*.*02)*	*(3*.*02)*	*(5*.*86)*
*MG*	*0*.069***	*0*.069***	*0*.069***
	*(14*.*22)*	*(14*.*23)*	*(8*.*43)*
*Betavals*	*0*.026***	*0*.026***	*0*.*026*
	*(5*.*80)*	*(5*.*81)*	*(1*.*23)*
*Tradeable*	*-0*.046***	*-0*.046***	*-0*.046**
	*(-5*.*54)*	*(-5*.*55)*	*(-2*.*96)*
*SOE*	*-0*.012**	*-0*.012**	*-0*.012***
	*(-2*.*41)*	*(-2*.*41)*	*(-11*.*77)*
*ESTAGE*	*-0*.028***	*-0*.028***	*-0*.028***
	*(-3*.*70)*	*(-3*.*70)*	*(-11*.*92)*
*BIG4*	*-0*.015*	*-0*.015*	*-0*.015***
	*(-1*.*88)*	*(-1*.*88)*	*(-5*.*08)*
*AUDITTYP*	*0*.167***	*0*.167***	*0*.167***
	*(11*.*49)*	*(11*.*49)*	*(17*.*50)*
*MPAY*	*0*.030***	*0*.030***	*0*.030***
	*(6*.*23)*	*(6*.*23)*	*(11*.*86)*
*Constant*	*-0*.824***	*-0*.736***	*-0*.824***
	*(-8*.*16)*	*(-7*.*07)*	*(-19*.*07)*
*Observations*	*16433*	*16433*	*16433*
*R* ^*2*^	*0*.*333*	*0*.*333*	*0*.*333*
*adj R* ^*2*^	*0*.*332*	*0*.*332*	*/*
*Within R* ^*2*^	*0*.*302*	*/*	*/*
*F*	*/*	*311*.*2*	*647*.*9*
*Industry FE*	*YES*	*YES*	*YES*
*Year FE*	*YES*	*YES*	*YES*

Note 1: t-statistics in parentheses.

Note 2

"***" denotes p<0.01

"**" denotes p<0.05, and

"*" denotes p<0.1, indicating statistical significance at the 1%, 5%, and 10% levels, respectively.

In the regression results of the three methods, the regression coefficients of *RPTs* are -0.019, which are significantly negative at the 1% level, providing evidence that supports hypothesis 1 of this paper that RPTs have a significant impact on the quasi-profit margin. This suggests that the method proposed in this paper, which uses the impact of RPTs on the quasi-profit margin to measure the effects of RPTs, is feasible.

In addition, the above evidence also verifies hypothesis 1–1 of this paper, which states that RPTs are significantly negatively correlated with the quasi-profit margin.

According to the regression results, it can be calculated that, ceteris paribus, for every 1% increase in the proportion of RPTs to the total assets at the beginning of the year, the quasi-profit margin of listed companies decreases by 0.019%. Based on the calculation formula of the quasi-profit margin (see Section “How to measure the effects of RPTs”), this implies that, under the same conditions, RPTs significantly reduce the operating profit margin or significantly increase the period expense ratio of listed companies. This further indicates that, in the Chinese A-share market, RPTs of listed companies overall exhibit a tunneling effect, which is consistent with the findings of existing literature. Furthermore, for financial regulatory authorities, it is imperative to continuously strengthen the identification and regulation of illegal RPTs, as this remains a long-term and arduous task.

#### The impact of transaction direction on the effects of RPTs

This article conducted regression analysis on Model (2) using LSDV, HDFE, and mixed regression with Driscoll-Kraay standard errors, and the regression results are shown in Table 6.

**Table 6:** Regression results of Model (2).

*Serials*	*(1)*	*(2)*	*(3)*
*Estimation Method*	*LSDV*	*HDFE*	*Driscoll-Kraay Pooled OLS*
*Model Type*	*Linear Model*		
*Std*. *Err*. *Type*	*Vce cluster (industry)*		*Driscoll-Kraay Std*. *Err*.
*Dependent Variable*	*QPM*		
*RPTs*	*-0*.029***	*-0*.029***	*-0*.029***
	*(-6*.*19)*	*(-6*.*19)*	*(-5*.*42)*
*SELLER*	*-0*.015***	*-0*.015***	*-0*.015***
	*(-4*.*52)*	*(-4*.*52)*	*(-6*.*93)*
*RPTs×SELLER*	*0*.026***	*0*.026***	*0*.026*
	*(4*.*00)*	*(4*.*00)*	*(2*.*42)*
*SIZE*	*0*.019***	*0*.019***	*0*.019***
	*(4*.*55)*	*(4*.*55)*	*(10*.*79)*
*LEV*	*-0*.293***	*-0*.293***	*-0*.293***
	*(-18*.*11)*	*(-18*.*12)*	*(-12*.*26)*
*TOP1*	*0*.114***	*0*.114***	*0*.114***
	*(5*.*01)*	*(5*.*01)*	*(16*.*39)*
*BALANCE*	*0*.013***	*0*.013***	*0*.013***
	*(2*.*94)*	*(2*.*94)*	*(5*.*58)*
*MG*	*0*.070***	*0*.070***	*0*.070***
	*(14*.*03)*	*(14*.*04)*	*(8*.*22)*
*Betavals*	*0*.026***	*0*.026***	*0*.*026*
	*(5*.*79)*	*(5*.*79)*	*(1*.*24)*
*Tradeable*	*-0*.046***	*-0*.046***	*-0*.046**
	*(-5*.*45)*	*(-5*.*45)*	*(-2*.*97)*
*SOE*	*-0*.012**	*-0*.012**	*-0*.012***
	*(-2*.*35)*	*(-2*.*35)*	*(-12*.*37)*
*ESTAGE*	*-0*.029***	*-0*.029***	*-0*.029***
	*(-3*.*72)*	*(-3*.*73)*	*(-11*.*85)*
*BIG4*	*-0*.015*	*-0*.015*	*-0*.015***
	*(-1*.*87)*	*(-1*.*87)*	*(-4*.*69)*
*AUDITTYP*	*0*.167***	*0*.167***	*0*.167***
	*(11*.*49)*	*(11*.*50)*	*(17*.*89)*
*MPAY*	*0*.030***	*0*.030***	*0*.030***
	*(6*.*27)*	*(6*.*27)*	*(12*.*80)*
*Constant*	*-0*.819***	*-0*.731***	*-0*.819***
	*(-8*.*06)*	*(-6*.*98)*	*(-21*.*92)*
*Observations*	*16433*	*16433*	*16433*
*R* ^*2*^	*0*.*334*	*0*.*334*	*0*.*334*
*adj R* ^*2*^	*0*.*333*	*0*.*333*	*/*
*Within R* ^*2*^	*0*.*303*	*/*	*/*
*F*	*/*	*285*.*5*	*1056*
*Industry FE*	*YES*	*YES*	*YES*
*Year FE*	*YES*	*YES*	*YES*

Note 1: t-statistics in parentheses.

Note 2

"***" denotes p<0.01

"**" denotes p<0.05, and

"*" denotes p<0.1, indicating statistical significance at the 1%, 5%, and 10% levels, respectively.

In the regression results of the three methods, the regression coefficients of *RPTs*,*SELLER*, and *RPTs*×*SELLER* are all significant, and the regression coefficients of *RPTs* and *SELLER* are significantly negative at the 1% level. The regression coefficient of *RPTs*×*SELLER* is significantly positive at the 1% or 10% level, indicating that *SELLER* has a significant negative moderating effect on the relationship between *RPTs* and *QPM*. These findings verify Hypothesis 2 of this article, which states that transaction direction have a significant impact on the effects of RPTs. They also confirm Hypothesis 2–1, which suggests that the greater the proportion of RPTs conducted from the seller’s perspective, the smaller the tunneling effect of RPTs. Furthermore, the above evidence strongly supports the buying inferior to selling theory proposed in this article.

Based on the marginal effect analysis derived from the regression results of the moderation effect model, the expression for the marginal effect of RPTs on quasi-profit margin is as follows:

marginaleffect=−0.029+0.026×SELLER

Ceteris paribus, for every 1% increase in the proportion of RPTs conducted by listed companies as sellers relative to total RPTs, the marginal effect of RPTs on the quasi-profit margin increases by 0.026%, indicating that the tunneling effect of RPTs decreases by 0.026%. This implies that the transaction direction has a significant impact on the effects of RPTs. Under the same conditions, the greater the proportion of RPTs conducted by listed companies as sellers, the smaller the overall tunneling effect of RPTs.

Combining the regression results and marginal effect analysis, it is evident that for listed companies, engaging more in RPTs as sellers can mitigate the tunneling effect of RPTs and reduce the risk of tunneling. For financial regulatory authorities, it is necessary to further refine the regulatory policies on RPTs. For instance, listed companies could be required to disclose RPTs information categorized by transaction role, with a particular focus on scrutinizing RPTs where the company acts as the buyer.

## Robustness tests

### Endogeneity issues

Although this paper conducts empirical analysis based on panel data and controls for year and industry fixed effects in model specification and regression analysis, endogeneity issues may still exist due to omitted variables and other reasons.

#### The effects of RPTs

(1) Model Design

To address potential endogeneity issues, this paper considers constructing the following simultaneous equation models and employs system estimation methods for regression analysis.


$${RPTs}_{it}=\alpha_{0}+{\alpha_{1}lnmeanRT}_{it}+{\alpha_{2}meanR20}_{it}+\alpha_{3}{SIZE}_{it}+\alpha_{4}{LEV}_{it}+\alpha_{5}{TOP1}_{it}+\alpha_{6}{BALANCE}_{it}+\alpha_{7}{MG}_{it}+\alpha_{8}{Betavals}_{it}+\alpha_{9}{Tradeable}_{it}+\alpha_{10}{SOE}_{it}+\alpha_{11}{ESTAGE}_{it}+\alpha_{12}{BIG4}_{it}+\alpha_{13}{AUDITTYP}_{it}+\alpha_{14}{MPAY}_{it}+{YEAR}_{FE}+{INDUSTRY}_{FE}+\varepsilon$$ Model (3-1)

$${QPM}_{it}=\alpha_{0}+{\alpha_{1}RPTs}_{it}+\alpha_{2}{SIZE}_{it}+\alpha_{3}{LEV}_{it}+\alpha_{4}{TOP1}_{it}+\alpha_{5}{BALANCE}_{it}+\alpha_{6}{MG}_{it}+\alpha_{7}{Betavals}_{it}+\alpha_{8}{Tradeable}_{it}+\alpha_{9}{SOE}_{it}+\alpha_{10}{ESTAGE}_{it}+\alpha_{11}{BIG4}_{it}+\alpha_{12}{AUDITTYP}_{it}+\alpha_{13}{MPAY}_{it}+{YEAR}_{FE}+{INDUSTRY}_{FE}+\varepsilon$$ Model (3-2)

In the equation, the subscript "*it*" represents the variable data of company *i* in year *t*,*YEAR*_*FE*_ and *INDUSTRY*_*FE*_ represent year and industry fixed effects, and *ε* represents the random error term.

In Model (3-1), two new variables are included as instrument variables for *RPTs*, namely *lnmeanRT* and *meanR2*0.

*lnmeanRT* represents the natural logarithm of the ratio of the annual total amount of RPTs to the annual number of RPTs.

The meaning and calculation method of *meanR*20 are as follows:

First, all samples are divided into 20 groups based on the quantile of the sum of the top ten shareholders’ shareholdings ratios, and each group is sequentially numbered from 01 to 20. Then, all samples are grouped by *YEAR*,*INDUSTRY*,*SOE*, and group number (01–20), and the group average of RPTs is calculated, which is denoted as *meanR*20.

This study examined the validity of these two instrumental variables (*lnmeanRT* and *meanR*20), and the test results indicate that both of these instrumental variables meet the requirements of relevance and exogeneity. For detailed test results, please refer to Online Appendix I.

(2) Regression Results Analysis

This paper uses two-stage least squares (2SLS), three-stage least squares (3SLS), and iterative three-stage least squares (I-3SLS) methods to estimate the simultaneous Eq Models (3-1) and (3-2). The regression results are shown in Table 7, where columns 2, 4, and 6 present the regression results of *RPTs* and control variables on *QPM*, and the regression coefficients of *RPTs* are significantly negative at the 1% level.

**Table 7:** Regression results of Model (3-1) to (3-2).

*Serials*	*(1)*	*(2)*	*(3)*	*(4)*	*(5)*	*(6)*
*Estimation Method*	*2SLS*		*3SLS*		*I-3SLS*	
*Model Type*	*Linear Model*					
*Std*. *Err*. *Type*	*Std*. *Err*.					
*Dependent Variable*	*RPTs*	*QPM*	*RPTs*	*QPM*	*RPTs*	*QPM*
*lnmeanRT*	*0*.191***		*0*.191***		*0*.191***	
	*(78*.*44)*		*(78*.*60)*		*(78*.*60)*	
*meanR20*	*0*.654***		*0*.654***		*0*.654***	
	*(53*.*05)*		*(53*.*19)*		*(53*.*19)*	
*RPTs*		*-0*.031***		*-0*.031***		*-0*.031***
		*(-9*.*20)*		*(-9*.*21)*		*(-9*.*21)*
*Observations*	*16433*	*16433*	*16433*	*16433*	*16433*	*16433*
*R* ^*2*^	*0*.*513*	*0*.*332*	*0*.*513*	*0*.*332*	*0*.*513*	*0*.*332*
*F*	*441*.*88*	*215*.*46*	*/*	*/*	*/*	*/*
*Control Variables*	*YES*	*YES*	*YES*	*YES*	*YES*	*YES*
*Industry FE*	*YES*	*YES*	*YES*	*YES*	*YES*	*YES*
*Year FE*	*YES*	*YES*	*YES*	*YES*	*YES*	*YES*

Note 1: t-statistics in parentheses.

Note 2: "***" denotes p<0.01

"**" denotes p<0.05, and

"*" denotes p<0.1, indicating statistical significance at the 1%, 5%, and 10% levels, respectively.

These findings suggest that:

1. Even from an endogenous perspective, both hypothesis 1 and hypothesis 1–1 in this paper hold, indicating that it is feasible to measure the effects of RPTs using their impact on the quasi-profit margin.
2. Based on the marginal effect analysis derived from the regression results, under the perspective of endogeneity, ceteris paribus, a 1% increase in the proportion of RPTs relative to the total assets at the beginning of the year will result in a 0.031% decrease in the quasi-profit margin of the listed company. This indicates that, all else being equal, RPTs significantly lower the operating profit margin or significantly increase the period expense rate of listed companies. It demonstrates that RPTs in China’s A-share listed companies exhibit an overall tunneling effect.
3. The regression coefficient of the core explanatory variable *RPTs* differs significantly between the endogenous and exogenous perspectives, indicating the presence of endogeneity issues. The regression coefficient of *RPTs* in the endogenous perspective (-0.031) is smaller than that in the exogenous perspective (-0.019), and the smaller the coefficient, the stronger the average tunneling effect of RPTs. Ignoring endogeneity issues would seriously underestimate the tunneling effect of RPTs.

#### The impact of transaction direction on the effects of RPTs

(1) Model Design

To address potential endogeneity issues, this paper considers constructing the following simultaneous equation models and employs system estimation methods for regression analysis.


$${RPTs}_{it}=\alpha_{0}+{\alpha_{1}lnmeanRT}_{it}+{\alpha_{2}meanR20}_{it}+\alpha_{3}{SIZE}_{it}+\alpha_{4}{LEV}_{it}+\alpha_{5}{TOP1}_{it}+\alpha_{6}{BALANCE}_{it}+\alpha_{7}{MG}_{it}+\alpha_{8}{Betavals}_{it}+\alpha_{9}{Tradeable}_{it}+\alpha_{10}{SOE}_{it}+\alpha_{11}{ESTAGE}_{it}+\alpha_{12}{BIG4}_{it}+\alpha_{13}{AUDITTYP}_{it}+\alpha_{14}{MPAY}_{it}+{YEAR}_{FE}+{INDUSTRY}_{FE}+\varepsilon$$ Model (4-1)

$${SELLER}_{it}=\alpha_{0}+{\alpha_{1}SELLER\_L}_{it}+\alpha_{2}{SIZE}_{it}+\alpha_{3}{LEV}_{it}+\alpha_{4}{TOP1}_{it}+\alpha_{5}{BALANCE}_{it}+\alpha_{6}{MG}_{it}+\alpha_{7}{Betavals}_{it}+\alpha_{8}{Tradeable}_{it}+\alpha_{9}{SOE}_{it}+\alpha_{10}{ESTAGE}_{it}+\alpha_{11}{BIG4}_{it}+\alpha_{12}{AUDITTYP}_{it}+\alpha_{13}{MPAY}_{it}+{YEAR}_{FE}+{INDUSTRY}_{FE}+\varepsilon$$ Model (4-2)

$${RPTs\times SELLER}_{it}=\alpha_{0}+{\alpha_{1}lnmeanRT\times SELLER\_L}_{it}+{\alpha_{2}meanR20\times SELLER\_L}_{it}+\alpha_{3}{SIZE}_{it}+\alpha_{4}{LEV}_{it}+\alpha_{5}{TOP1}_{it}+\alpha_{6}{BALANCE}_{it}+\alpha_{7}{MG}_{it}+\alpha_{8}{Betavals}_{it}+\alpha_{9}{Tradeable}_{it}+\alpha_{10}{SOE}_{it}+\alpha_{11}{ESTAGE}_{it}+\alpha_{12}{BIG4}_{it}+\alpha_{13}{AUDITTYP}_{it}+\alpha_{14}{MPAY}_{it}+{YEAR}_{FE}+{INDUSTRY}_{FE}+\varepsilon$$ Model (4-3)

$${QPM}_{it}=\alpha_{0}+{\alpha_{1}RPTs}_{it}+\alpha_{2}{SELLER}_{it}+\alpha_{3}{RPTs\times SELLER}_{it}+\alpha_{4}{SIZE}_{it}+\alpha_{5}{LEV}_{it}+\alpha_{6}{TOP1}_{it}+\alpha_{7}{BALANCE}_{it}+\alpha_{8}{MG}_{it}+\alpha_{9}{Betavals}_{it}+\alpha_{10}{Tradeable}_{it}+\alpha_{11}{SOE}_{it}+\alpha_{12}{ESTAGE}_{it}+\alpha_{13}{BIG4}_{it}+\alpha_{14}{AUDITTYP}_{it}+\alpha_{15}{MPAY}_{it}+{YEAR}_{FE}+{INDUSTRY}_{FE}+\varepsilon$$ Model (4-4)

In these equations, the subscript "*it*" denotes the variable data of company *i* in year *t*,*YEAR*_*FE*_ and *INDUSTRY*_*FE*_ denote the fixed effects of year and industry, respectively, and *ε* represents the random error term.

*SELLER*_*L* represents the lagged data of *SELLER* by one period and serves as an instrumental variable for *SELLER*. Analysis reveals that *SELLER*_*L*, as the previous period’s data for *SELLER*, is closely related to *SELLER*. However, *SELLER*_*L* does not seem to have a direct impact on the dependent variable *QPM* in this study. Therefore, theoretically, using *SELLER*_*L* as an instrumental variable for *SELLER* is reasonable to some extent.

(2) Regression Results Analysis

This paper employs the Iterative Three-Stage Least Squares (I-3SLS) method to regress the simultaneous Eq Models (4-1) ~ (4-4), and the regression results are shown in Table 8. The fourth column presents the regression results of *RPTs*,*SELLER*,*RPTs*×*SELLER*, and control variables on *QPM*. The regression coefficients of *RPTs*,*SELLER*, and *RPTs*×*SELLER* are all significant, and the regression coefficients of *RPTs* and *SELLER* are significantly negative at the 1% level, while the regression coefficient of *RPTs*×*SELLER* is significantly positive at the 1% level.

**Table 8:** Regression results of Model (4-1) to (4-4).

*Serials*	*(1)*	*(2)*	*(3)*	*(4)*
*Estimation Method*	*I-3SLS*			
*Model Type*	*Linear Model*			
*Std*. *Err*. *Type*	*Std*. *Err*.			
*Dependent Variable*	*RPTs*	*SELLER*	*RPTs×SELLER*	*QPM*
*lnmeanRT*	*0*.115***			
	*(57*.*95)*			
*meanR20*	*0*.617***			
	*(47*.*97)*			
*SELLER_L*		*0*.576***		
		*(78*.*33)*		
*lnmeanRT×SELLER_L*			*0*.001***	
			*(3*.*26)*	
*meanR20×SELLER_L*			*0*.577***	
			*(37*.*95)*	
*RPTs*				*-0*.057***
				*(-6*.*41)*
*SELLER*				*-0*.037***
				*(-3*.*83)*
*RPTs×SELLER*				*0*.060***
				*(2*.*94)*
*Observations*	*12677*	*12677*	*12677*	*12677*
*R* ^*2*^	*0*.*468*	*0*.*348*	*0*.*210*	*0*.*324*
*Control Variables*	*YES*	*YES*	*YES*	*YES*
*Industry FE*	*YES*	*YES*	*YES*	*YES*
*Year FE*	*YES*	*YES*	*YES*	*YES*

Note 1: t-statistics in parentheses.

Note 2

"***" denotes p<0.01

"**" denotes p<0.05, and

"*" denotes p<0.1, indicating statistical significance at the 1%, 5%, and 10% levels, respectively.

These pieces of evidence indicate that:

1. Even from an endogenous perspective, *SELLER* still has a significant negative moderating effect on the relationship between *RPTs* and *QPM*. Therefore, Hypothesis 2 and Hypothesis 2–1 in this paper are still valid, further verifying the buying inferior to selling theory proposed in this paper.
2. Based on the marginal effect analysis under the perspective of endogeneity, the marginal effect of RPTs on the quasi-profit margin is expressed as:
marginaleffect=−0.057+0.06×SELLER
  Ceteris paribus, for each 1% increase in the proportion of RPTs conducted by the listed company as the seller, the marginal effect on the quasi-profit margin increases by 0.06%, meaning the tunneling effect of RPTs decreases by 0.06%. This implies that, all else being equal, the higher the proportion of RPTs in which the listed company acts as the seller, the smaller the tunneling effect of the RPTs. For listed companies, conducting more RPTs as the seller can suppress the tunneling effect of RPTs and reduce the associated risks.
3. The regression coefficients of the core explanatory variable *RPTs* and the moderating variable *SELLER* differ significantly between the endogenous and exogenous perspectives, indicating the existence of endogeneity issues. Ignoring endogeneity issues may lead to incorrect estimates of the impact of transaction direction on the effects of RPTs.

### Robustness tests

The previous section empirically tested the effects of RPTs and the impact of transaction direction on the effects of RPTs from both an exogenous and endogenous perspective. Although there were some differences in the regression results between the two perspectives, both supported the hypotheses proposed in this paper. In other words, conducting regression analysis from an endogenous perspective can be considered a robustness check of the regression results obtained from an exogenous perspective to some extent.

To ensure the robustness of the results, this paper also conducted robustness tests by modifying the sample time span and replacing variable indicators.

#### The method of modifying sample time span

The original time span of the sample is from 2016 to 2021. Based on this, the sample data from 2017 to 2020 is extracted for robustness testing using models (4–1) to (4–4). The regression results for *RPTs*,*SELLER*,*RPTs*×*SELLER* and control variables on *QPM* are shown in Table 9, where the fourth column presents the regression results. The regression coefficients of *RPTs*,*SELLER*, and *RPTs*×*SELLER* are all significant, with *RPTs* and *SELLER* both significantly negative at the 1% level, and *RPTs*×*SELLER* significantly positive at the 1% level. These results suggest that the findings of this study are robust.

**Table 9:** Robustness test results (based on sample data from 2017 to 2020).

*Serials*	*(1)*	*(2)*	*(3)*	*(4)*
*Estimation Method*	*I-3SLS*			
*Model Type*	*Linear Model*			
*Std*. *Err*. *Type*	*Std*. *Err*.			
*Dependent Variable*	*RPTs*	*SELLER*	*RPTs×SELLER*	*QPM*
*lnmeanRT*	*0*.116***			
	*(50*.*10)*			
*meanR20*	*0*.619***			
	*(41*.*30)*			
*SELLER_L*		*0*.568***		
		*(67*.*44)*		
*lnmeanRT×SELLER_L*			*0*.002***	
			*(3*.*10)*	
*meanR20×SELLER_L*			*0*.554***	
			*(31*.*61)*	
*RPTs*				*-0*.063***
				*(-6*.*06)*
*SELLER*				*-0*.048***
				*(-4*.*14)*
*RPTs×SELLER*				*0*.088***
				*(3*.*59)*
*Observations*	*9757*	*9757*	*9757*	*9757*
*R* ^*2*^	*0*.*463*	*0*.*342*	*0*.*202*	*0*.*319*
*Control Variables*	*YES*	*YES*	*YES*	*YES*
*Industry FE*	*YES*	*YES*	*YES*	*YES*
*Year FE*	*YES*	*YES*	*YES*	*YES*

Note 1: t-statistics in parentheses.

Note 2

"***" denotes p<0.01

"**" denotes p<0.05, and

"*" denotes p<0.1, indicating statistical significance at the 1%, 5%, and 10% levels, respectively.

#### The method of replacing variable indicators

The previous section measured the transaction direction of listed companies in RPTs by the ratio of the amount of RPTs conducted by the listed companies as sellers to the total amount of RPTs, and it was denoted as *SELLER*. Here, we consider using the difference between the proportions of RPTs by listed companies as sellers and buyers, respectively, to measure the transaction direction, and it can be denoted as *DIFFER*. Based on models (4–1) ~ (4–4), robustness tests were conducted and the regression results are shown in Table 10. The fourth column shows the regression results of *RPTs*,*DIFFER*,*RPTs*×*DIFFER*, and control variables on *QPM*. The regression coefficients of *RPTs*,*DIFFER*, and *RPTs*×*DIFFER* are all significant, and the regression coefficients of *RPTs* and *DIFFER* are significantly negative at the 1% level, while the regression coefficient of *RPTs*×*DIFFER* is significantly positive at the 1% level. This further demonstrates the robustness of the research results.

**Table 10:** Robustness test results (Using *DIFFER* to measure transaction direction).

*Serials*	*(1)*	*(2)*	*(3)*	*(4)*
*Estimation Method*	*I-3SLS*			
*Model Type*	*Linear Model*			
*Std*. *Err*. *Type*	*Std*. *Err*.			
*Dependent Variable*	*RPTs*	*DIFFER*	*RPTs×DIFFER*	*QPM*
*lnmeanRT*	*0*.187***			
	*(68*.*77)*			
*meanR20*	*0*.640***			
	*(43*.*21)*			
*DIFFER_L*		*0*.577***		
		*(78*.*90)*		
*lnmeanRT×DIFFER_L*			*0*.002***	
			*(4*.*52)*	
*meanR20×DIFFER_L*			*0*.466***	
			*(24*.*30)*	
*RPTs*				*-0*.029***
				*(-6*.*19)*
*DIFFER*				*-0*.017***
				*(-3*.*64)*
*RPTs×DIFFER*				*0*.031***
				*(3*.*02)*
*Observations*	*12677*	*12677*	*12677*	*12677*
*R* ^*2*^	*0*.*500*	*0*.*357*	*0*.*194*	*0*.*324*
*Control Variables*	*YES*	*YES*	*YES*	*YES*
*Industry FE*	*YES*	*YES*	*YES*	*YES*
*Year FE*	*YES*	*YES*	*YES*	*YES*

Note 1: t-statistics in parentheses.

Note 2

"***" denotes p<0.01

"**" denotes p<0.05, and

"*" denotes p<0.1, indicating statistical significance at the 1%, 5%, and 10% levels, respectively.

Note 3: DIFFER_L represents the lagged data of DIFFER by one period.

## Conclusions and discussions

### Key findings

This paper first conducts theoretical analysis of the two effects of RPTs, namely the efficiency-enhancing effect and the tunneling effect, based on the transaction cost theory and the information asymmetry theory. Then constructs a new indicator, the quasi-profit margin, to assess the effects of RPTs by studying their impact on the quasi-profit margin. Based on the asymmetry of information between the two parties involved in the transaction and the theory of information screening, the buying inferior to selling theory is proposed, systematically explaining the impact of transaction direction on the effects of RPTs. Subsequently, fixed-effects models are constructed using panel data of Chinese A-share listed companies from 2016 to 2021. Empirical research is conducted from both exogeneity and endogeneity perspectives, employing estimation methods such as high dimensional fixed effects method, two-stage least squares method, and three-stage least squares method. The research results show that:

1. Under ceteris paribus conditions, RPTs significantly reduce the quasi-profit margin of listed companies.
2. Under ceteris paribus conditions, the transaction direction significantly moderates the relationship between RPTs and the quasi-profit margin, meaning the transaction direction significantly impacts the effects of RPTs. The greater the proportion of RPTs conducted by the listed company as sellers, the smaller the overall tunneling effect of the RPTs.

### Significance and potential contribution

1. This paper proposes a novel method to evaluate the effects of RPTs by examining the impact of RPTs on the quasi-profit margin. The results indicate that RPTs in Chinese A-share listed companies generally exhibit a tunneling effect, which is consistent with existing literature. This also indirectly reflects the need for financial regulatory authorities to continuously enhance the identification and rectification of irregular RPTs.
2. This paper primarily investigates the impact of transaction direction on the effects of RPTs, a perspective that has been overlooked in existing literature. The study enriches the related research literature, expands the theoretical framework of factors influencing the effects of RPTs, and provides some insights for listed companies and financial regulatory authorities. For listed companies, engaging in RPTs more as sellers can mitigate the tunneling effect and reduce tunneling risks. For financial regulatory authorities, there is a need to further refine the control policies for RPTs. For instance, they could require listed companies to disclose RPTs information categorized by transaction role and focus on scrutinizing RPTs where the company acts as the buyer.

### Limitations of this study

1. This study conducted empirical research based on micro-level data of listed companies, without taking into account macro-level factors such as government-market relations, economic development environment, legal system environment, and marketization index in model design and control variable selection. Nevertheless, the research findings of this study are still reliable. The reasons are as follows: Firstly, the research objects of this study are listed companies in China’s A-share market, and the environmental conditions of all sample companies at the macro-level are basically consistent. Secondly, this study controlled for year and industry fixed effects in model design and regression analysis. These two fixed effects to some extent represent the unquantifiable macro-level environmental conditions that are only related to the year or industry but not to the sample individuals. Thirdly, this study used simultaneous equation models and system estimation methods to deal with endogeneity issues, which to some extent avoided the interference of omitted variables on the research results.
2. This study elaborated on the buying inferior to selling theory from three dimensions: a single RPT, multiple RPTs, and coexistence of RPTs and non-RPTs. However, due to the limitations of the research topic, this study did not empirically test the theory of "buying inferior to selling" in the dimension of coexistence of RPTs and non-RPTs.

### Future research directions

1. RPTs have the tunneling effect, but cannot be directly equated with tunneling. in future related research, it is recommended that scholars comprehensively understand and objectively evaluate the effects of RPTs.
2. Following the theoretical framework presented in this paper, an interesting observation may be arise that tunneling behavior by majority shareholders though RPTs can be classified into two types: active and passive. Active tunneling behavior means when the majority shareholders of a listed company have both the ability and motive to extract value and does so by engaging in undisclosed, unfair RPTs to transfer the listed company’s interests to related parties. Passive tunneling behavior means when the controlling shareholder of a listed company does not have the intention to extract value from the listed company but instead seeks to achieve a win-win situation through RPTs with related parties in order to improve the performance of the listed company, but the interests of the listed company are silently flowing to the related parties through regular RPTs. This is because the traders overlook the profound impact of the transaction direction on the effects of RPTs.
3. Validating the buying inferior to selling theory presented in this paper in the dimension where RPTs and non-RPTs coexist is an interesting and challenging task. Scholars who are interested are welcome to conduct research on this topic together.
